# Iowa State Veterinary Medical Association

**Published:** 1897-04

**Authors:** 


### IOWA STATE VETERINARY MEDICAL ASSOCIATION.

(Reported for the JOURNAL by our Correspondent.)

THE Association held its ninth annual meeting in the Capitol Building at Des Moines, Ia., on Wednesday and Thursday, January 13 and 14, 1897.

About thirty members were present, which was a large attendance, considering the condition of the profession in this State at the present time.

First Day: Dr. L. U. Shipley, President, delivered an interesting address touching on the present condition of the profession in general and that of Iowa in particular, and offered some valuable suggestions relative to legislation and sanitation.

The Committees on Stock Paper Publications and Army Legislation failed to present any reports, but a resolution was adopted asking the Iowa members of Congress to support the bill of the Army Legislative Committee of the U. S. V. M. A.

Another resolution was adopted urging our Congressmen to do all in their power to defeat the pending bill relative to vivisection in the District of Columbia.

A number of new members were admitted.

The Committee on State Legislation reported that they had secured the introduction of a bill controlling the use of the title of veterinarian, but that they were unable to secure its enactment.

The Committee on Sanitation presented a report touching on the state of milk- and meat-inspection within the State and the larger cities of the United States, a short synopsis of the laws of the various States relative to the control of contagious diseases among live-stock, and a short *résumé* of the bills considered by the State Legislature at its last session relative to veterinary sanitation.

An invitation was extended to the State Fair delegates who were in session in another portion of the building to meet with us after supper and listen to a paper on "Serum-therapy in Hog-cholera," by Dr. A. F. Peters, of Lincoln, Nebraska. The invitation was accepted and at the appointed time and place Dr. Shipley called the two Associations to order.

Dr. Peters's paper brought out an animated discussion that was cut short by the President-elect of the Fair Association stating that it was time for the members of that Association to withdraw to participate in their annual banquet, and who extended an invitation to the members of the Veterinary Association to partake of their hospitality at the banquet table. On motion, this invitation was accepted. Most of the members of both Associations, to the number of about 125, repaired to the banquet hall, where, after feasting the physical man, we were entertained until a late hour by a number of carefully prepared and spicy toasts.

Second Day: President Shipley called the Association to order promptly at 9 A.M. The business transacted was the election of officers for the ensuing year, which resulted as follows: President, G. A. Johnson, of Sioux City; First Vice-President, S. H. Kingery, of Creston; Second Vice-President, J. H. McLeod, of Charles City; Secretary and Treasurer, J. E. Brown, of Oskaloosa.

The President then introduced Dr. J. F. Kennedy, Secretary of the Iowa State Board of Health, who read a very able paper on the "Relation of the Veterinary Profession to the Public Health."

Prof. W. B. Niles delivered a short lecture on "Parasitic Disease of Sheep." President Shipley reported a case where hogs contracted tuberculosis from a tuberculous cow. The following papers were then presented: "Duties of the State Veterinary Surgeon," by Dr. J. I. Gibson, State Veterinarian; "Ergot in the Treatment of Fungoid Growths," by Dr. W. A. Heck; "Azoturia," by Dr. W. H. Austin; "Thermo-cautery in Veterinary Practice," by Dr. P.O. Koto; "Lymphoma," by Dr. Miller; "Barium Chloride: Its Uses in Colic," by Dr. J. E. Brown. Each of these papers provoked an animated discussion, which, owing to the lack of time, had to be cut short in some cases.

At 6.15 P.M. the meeting adjourned, to meet at such time and place as the President and Secretary should designate.

Dr. Peters's (of Lincoln, Neb.) good work lent much interest to the meeting, and it was with much pleasure that the Association unanimously elected him an honorary member.

To those members of the Association who failed to attend we extend our sympathies, for they certainly missed a rare treat, both from an instructive and social point of view. One of the very pleasing features of this was the acquaintance and fraternal feeling established between the State Fair and Iowa State Veterinary Medical Associations.

The State Dairy Commission was in attendance a part of the time during both days' sessions, and took an active part in the discussions, especially those relative to milk-inspection and tuberculosis.

This closed one of the best meetings ever held by any State association in this country, and we sincerely hope that others as good may follow, and that none of the members will be compelled to miss the benefits that are to be derived from an attendance upon such meetings.

### MASSACHUSETTS VETERINARY MEDICAL ASSOCIATION.

